# Cell-Mediated Immunoreactivity of Poly(2-isopropenyl-2-oxazoline) as Promising Formulation for Immunomodulation

**DOI:** 10.3390/ma14061371

**Published:** 2021-03-12

**Authors:** Ema Paulovičová, Zuzana Kroneková, Lucia Paulovičová, Monika Majerčíková, Juraj Kronek

**Affiliations:** 1 Immunol & Cell Culture Laboratories, Department Immunochemistry of Glycoconjugates, Center of Glycomics, Institute of Chemistry, Slovak Academy of Sciences, Dúbravská cesta 9, 845 38 Bratislava, Slovakia; ema.paulovicova@savba.sk (E.P.); lucia.paulovicova@savba.sk (L.P.); 2 Department for Biomaterials Research, Polymer Institute, Slovak Academy of Sciences, Dúbravská cesta 9, 845 41 Bratislava, Slovakia; zuzana.kronekova@savba.sk (Z.K.); upolmoma@savba.sk (M.M.)

**Keywords:** poly(2-isopropenyl-2-oxazoline), immunomodulation, cytokines, RAW 264.7, phagocytosis, cell internalization

## Abstract

Poly(2-isopropenyl-2-oxazoline) (PIPOx) represents a functional polymer with high potential for drug delivery, tissue engineering, and immunomodulation. The immunomodulatory efficiency of the PIPOx formulation has been studied in vitro following splenic cells and RAW 264.7 macrophages exposition. The cell-specific immunomodulative effect on production of Th1, Th2, Th17, and Treg signature cytokines has been demonstrated. The impact on the functionality of PIPOx-sensitized RAW 264.7 macrophages was assessed by cell phagocytosis. Time- and concentration-dependent cell internalization and intracellular organelles colocalization of fluorescently labeled PIPOx has been examined. The in vitro results demonstrated the PIPOx bioavailability and the capability of triggering immune cell responses resulting in the induced production of cell-specific signature interleukins, important prerequisite properties for future potential biomedical applications.

## 1. Introduction

Poly(2-alkyl-2-oxazolines) represent biocompatible and non-cytotoxic polymeric materials [1] with a high potential in different biomedical applications such as controlled drug and gene release, tissue engineering, hydrogel technologies, etc. [2,3,4]. Usually, they are achieved in the cationic ring-opening polymerization (CROP) of 2-alkyl-2-oxazolines leading to polymers with defined structure, predicted molar mass, and narrow dispersity [5,6]. Using different 2-oxazoline monomers, the wide library of hydrophilic, thermosensitive [7,8], hydrophobic, amphiphilic [9], or functional [10] polymers has been prepared. Apart from 2-alkyl-2-oxazolines, 2-alkenyl-2-oxazolines can provide, depending on polymerization conditions, several polymerization reactions leading to polymers with various structures and architectures [11,12,13,14].

From this group of monomers, 2-isopropenyl-2-oxazoline represents a monomer with dual orthogonal functionality able to polymerize by CROP of 2-oxazoline unit providing polymers with free double bonds [15,16]. On the other hand, an isopropenyl unit enables the preparation of poly(2-isopropenyl-2-oxazolines) (PIPOx) containing a free 2-oxazoline group in the side chain. Free-radical polymerization belongs to the most common polymerizations of 2-isopropenyl-2-oxazolines leading to polymers with broader dispersity (Đ) and lower control over molar mass and architecture [17,18]. Similarly, polymers with broader dispersity were achieved in the frustrated Lewis-pair polymerization of 2-isopropenyl-2-oxazoline [19]. Better-defined polymers have been achieved using various methods of living or controlled polymerizations. Living polymerizations of 2-isopropenyl-2-oxazoline typically involve living anionic polymerization initiated by diphenylmethylpotassium/diethylzinc [20] or n-butyllithium [21]. Polymers with low dispersity were also achieved in the rare-earth metal-mediated group transfer polymerization of 2-isopropenyl-2-oxazoline [22]. On the other hand, reversible addition−fragmentation chain transfer polymerization of 2-isopropenyl-2-oxazoline provided PIPOx with molar mass under 3000 g/mol and relatively high dispersity around 1.35 [23]. Moreover, polymerizations proceeded in only 30% of conversions. Recently, Raus at al. for the first time successfully prepared PIPOx through aqueous Cu(0)-mediated atom-transfer radical polymerization of 2-isopropenyl-2-oxazoline in a controlled way initiated and catalyzed by the 2-chloropropionitrile/CuCl(CuCl_2_)/TPMA system [24].

PIPOx containing a free 2-oxazoline ring can be further modified employing a reaction of 2-oxazoline moiety with compounds containing thiol [25] or carboxylic groups [17,18,23,25]. Due to these unique chemical features, PIPOx is currently attractive for different fields of biotechnology and medicine due to possible preparation of thermosensitive polymers [26,27], hydrogels [27,28,29,30], or (bio)conjugates with peptides, saccharides, or drugs [31]. Therefore, their rising importance is expected, especially in drug delivery, gene delivery, tissue engineering, or vaccine technology. The basic requirement for the perspective use in biomedical applications is the tolerance of cells to the used polymer materials. Therefore, the assessment of biocompatibility using tissue cultures evaluating acute and system toxicity, examining of the inhibition of cell growth, mutagenicity, carcinogenicity, teratogenicity, and pro-allergenic potential must be included.

Immunomodulative activities of polymeric biomaterials have become the most relevant ones concerning their bioavailability and biocompatibility. Different strategies of triggering the appropriate immune system responses by functional biomaterials and various applications of biomaterials mimicking the physiological extracellular matrix and modifications of cell-mediated immune responses are of interest [32,33]. Spleen-derived cells are represented by a mixture of immune cells including macrophages, monocytes, dendritic cells (DCs), and T-lymphocytes that possess different functions in immune system. Myeloid phagocytes such as macrophages or DCs representing a complex network of cells with protective functionalities are also involved in mechanisms of homoeostasis such as tissue remodeling and wound healing [34]. Macrophages, comparable with Th-lymphocytes, have been divided into main subsets: pro-inflammatory M1-classically activated macrophages and anti-inflammatory M2-alternatively activated macrophages with distinct functional and phenotypical characteristics [35,36]. In general, inflammatory cytokines such as TNF-α and IFN-γ induce the M1 phenotype. On the contrary, anti-inflammatory interleukins such as IL-10, IL-4, and IL-13 induce the M2 phenotype [33]. Macrophages play an important role in orchestrating immune responses to biomaterials used in the construction of implantable devices and drug-delivery systems [37].

It was shown recently that positively charged polymers such as polyethyleneimine and cationic dextran have the potential to modulate macrophages and change their phenotype from tumor growth-promoting M2 macrophages to anti-tumor M1 macrophages [38]. In the last study, we showed that PIPOx is also not cytotoxic to the cells up to 10 mg/mL and significantly stimulates in vitro and ex vivo proliferation of macrophages [18]. We have shown that co-stimulation of non-adherent cells (T-lymphocyte-enriched splenocytes) with PIPOx-stimulated adherent cells (enriched in DCs) leads to their induced proliferation. These results suggest that PIPOx may play a role in various immunomodulatory processes [18].

In this work, we focused on the cell-mediated bioimmunological behavior of PIPOx and its cellular compatibility as promising matrix biomaterial. The sequential adherence of splenocytes and isolated cell populations have been used to ascertain their immunobiological activity following PIPOx exposure. The aim of study suggested the sensitization of population of adherent CD11c^+^ and CD14^+^ spleen cells and adherent spleen cells more enriched in CD11c^+^ antigen-presenting cells with PIPOx and follow up the polarization of immune response towards Th1, Th2, Th17, or Treg evaluated by measuring the production of selected signature cytokines. The cytotoxicity has been determined via phagocytosis process. Next, the RAW 264.7 macrophage internalization of PIPOx-FITC followed by intracellular localization has been assayed based on colocalization in vesicular structures of the cells resembling organelles of phagocytic and/or endocytic pathway.

## 2. Materials and Methods

### 2.1. Material

2-Isopropenyl-2-oxazoline (Sigma-Aldrich, Weinheim, Germany) was distilled in the presence of 2,6-di-tert-butyl-4-methylphenol (Sigma-Aldrich, Weinheim, Germany) under reduced pressure, and stored at −20 °C under argon atmosphere. N,N-Dimethylacetamide (DMAc) (Sigma-Aldrich, Weinheim, Germany) was distilled over phosphorus pentaoxide (Sigma-Aldrich, Weinheim, Germany) under reduced pressure. Fluorescein isothiocyanate (FITC) and dimethylsulfoxide (DMSO) were purchased from Sigma-Aldrich (Weinheim, Germany) and used as received. 4-Aminobutyric acid (ABA) was purchased from Fluka (Buchs, Germany) and used as received.

### 2.2. Synthesis of Polymers

PIPOx was synthesized by a free-radical polymerization in bulk initiated by azobisisobutyronitrile (AIBN) as described elsewhere [18]. Molar mass of PIPOx was equal to 21,000 g/mol and Đ = 1.85. For ^1^H nuclear magnetic resonance (NMR) and Fourier-transform infrared spectroscopy (FTIR) spectra of PIPOx see Figures S1 and S2 in Supplementary Material.

PIPOx labeled with FITC (PIPOx-FITC) was prepared from PIPOx by two-step synthesis. In the first step, PIPOx (0.22 g, 0.001 mol of structural units) and ABA (0.020 g, 0.0002 mol) were dissolved in dry DMAc (1 mL) and heated at 120 °C for 6 h under argon. Reaction mixture was cooled, diluted with 5 mL of water, dialyzed towards deionized water (dialysis membrane Spectra/Por 6, Molecular weight cut-off (MWCO) 3.5 kDa, Spectrum Laboratories, Rancho Dominguez, CA, USA) for 4 × 1.5 h, and freeze-dried. Yield of reaction was 95% of PIPOx containing ABA (PIPOx-ABA). The chemical structure was determined from FTIR and NMR (Figures S3 and S2 in Supplementary Material). Prepared intermediate was functionalized with FITC using a standard protocol for labeling proteins. Briefly, 23 mg of PIPOx-ABA was dissolved in 0.1 M bicarbonate buffer (10 mL, pH = 9) and 1.6 mL of FITC in DMSO (1 mg/mL) was dropwise added for 5 min. Reaction mixture was held in 2–8 °C for 8 h under argon and product was purified using dialysis toward distilled water (dialysis membrane Spectra/Por 6, MWCO 3.5 kDa, Spectrum Laboratories, Rancho Dominguez, CA, USA). Yield: ~100%. The chemical structure was evaluated using ^1^H NMR and FTIR (Figures S4 and S2 in Supplementary Material), and degree of addition was calculated from UV/Vis absorption spectroscopy (Figure S5 in Supplementary Material).

### 2.3. Analytical Methods

The ^1^H and ^13^C NMR spectra for all polymers were conducted in CDCl_3_ and DMSO-d_6_ at room temperature using a Varian VXR-400 (Wilmington, DE, USA). In all measurements, tetramethylsilane was used as an internal standard. ATR-FTIR spectra of polymers were conducted by NICOLET 8700 (Thermo Scientific, Madison, WI, USA) using a Ge crystal with 64 scans and in resolution of 4 cm^−^^1^.

The molar mass and dispersity of PIPOx was recorded using size exclusion chromatography (SEC) as described elsewhere [18].

The UV/VIS spectra were recorded using Shimadzu 1650 PC (Shimadzu, Kyoto, Japan) in methanol at the concentration of 0.1 mg/mL for labeled polymers in the range of 210 to 600 nm in the resolution of 0.2 nm. The emission spectra were recorded on a Shimadzu RF-5301 (Shimadzu, Kyoto, Japan) in methanol using concentration ranged from 0.01 to 0.1 mg/mL with excitation wavelength of 495 nm.

### 2.4. Preparation of Splenocytes

Balb/c mice (female, 8–12 weeks old, breeding facility VELAZ, Prague, Czech Republic) were used for extirpation of spleens and isolation of splenocytes. The experiments were performed according to GLP and OECD guidelines, based on the ethical guidelines of the Research Base of Slovak Medical University, Institute of Preventive and Clinical Medicine (Bratislava, Slovakia), the approval No. Ro 2939/09-221 of State veterinary and food administration of the Slovak Republic. Spleens were aseptically removed and were poured into the ice-cold saline (1 mL per spleen). Splenocytes were isolated by homogenization of splenic tissue with the plunger end of the syringe. The splenocytes suspension was filtered (50μm-mesh filter (CellTrics disposable filter; Partec, Görlitz, Germany) and centrifuged at 800× *g* for 10 min at 4 °C. The splenocytes were resuspended in 5 mL of ACK lysis buffer (0.15 M NH_4_Cl, 1 M K_2_CO_3_, and 0.01 M EDTA, pH 7.2) and the lysis of erythrocytes was completed at room temperature for 5 min. Afterwards, cells were washed twice with saline and resuspended in complete RPMI-1640 medium (Lonza, Basel, Switzerland) supplemented with 10 % of fetal bovine serum, penicillin (100 U/mL) and streptomycin sulphate (100 mg/mL) (Gibco, NY, USA). Following assessment of splenocyte viability by Trypan blue staining method, the density of cells has been adjusted to 1 × 10^6^ cells/mL.

### 2.5. Stimulation

Isolated and collected splenocytes were seeded (4 × 10^5^ cells per well) into 24 well culture plates (Nunc, Roskilde, Denmark) and stimulated in vitro with Concanavalin A (Con A), final concentration of 10 µg/mL (Sigma, Stockholm, Sweden) and PIPOx (final concentration of 5 mg/mL) for 24 h in a 37 °C incubator (5% CO_2_, humidified atmosphere). Following the exposition, the culture media were stored at −20 °C for determination of interleukins and growth factors.

Isolated splenocytes in complete RPMI-1640 (1 × 10^6^ cells per ml) were seeded into 6-well culture plates (Nunc, Denmark). Afterwards, the cells were incubated for 1 h at 37 °C (humidified incubator, 5% CO_2_). Following this 1st incubation period, non-adherent cells were taken away and seeded into new 6-well culture plates. The adherent cells from the 1st incubation period were washed 3 times to take out all non-adherent cells and were adjusted with 500 µL of fresh complete RPMI-1640 medium. The first isolated non-adherent cells were allowed to adhere overnight in the complete RPMI-1640 medium at 37 °C, 5% CO_2_ in humidified incubator. Following this 2nd adherence period splenocytes depleted of adherent cells were collected and the adherent cells after 2nd adherence period were washed 3 times to eliminate any non-adherent cells.

The adherent cells (following the 1st and the 2nd incubation period) were harvested. Adherent cells obtained in this fashion after the 1st and the 2nd adherence period together with non-adherent cells were subjected to the immunocytometric analysis (FC500, Beckman Coulter, Fullerton, CA, USA) using Anti-Mouse CD3-FITC (clone KT3, Rat IgG2a), Anti-Mouse CD4-PE (clone YTS, Rat IgG2b), Anti-Mouse CD8α-PE (clone KT15, Rat IgG2a), Anti-Mouse CD11c-FITC (clone N418, Armenian Hamster IgG) (Antigenix America Inc., Melville, NY, USA), Anti-Mouse CD14-PE (clone rmC5-3 (RUO), Rat LOU/M IgG1, κ, BD Pharmingen). Phenotyping analysis of adherent cells after the 1st adherence period revealed enrichments in DCs and monocytes/macrophages with reduced proportion of lymphocytes (Table 1). Adherent cells obtained after the 2nd adherence period were more enriched in DCs with fewer monocytes/macrophages in comparison with enrichment after the 1st adherence period, as resulted from the immunocytometric assay of CD11c^+^ and CD14^+^ immunocytes.

The adherent and non-adherent cells from sequential adherent phases underwent in vitro stimulation with Con A and PIPOx in the same way as previous stimulation of splenocytes was conducted. Co-stimulation of adherent cells (1 × 10^6^ cells/mL, adherent cells after the 1st and the 2nd adherence period, unstimulated or stimulated with Con A and PIPOx) with non-adherent splenocytes (1 × 10^6^ cells/mL) was carried out by co-cultivation in fresh complete RPMI-1640 medium for 4 days (37 °C humidified incubator, 5% CO_2_ atmosphere).

### 2.6. Cytokine Secretion Assay, Immunocytometry, and ELISA

As previously described, washed and lysed splenocytes were adjusted by growth medium approximately to 4 × 10^7^ cell/mL and 400 μL aliquots were stimulated with PIPOx (concentration of 5 mg/mL) and plated in 24-well tissue culture plates (Nunc, Denmark). Polyclonal cell stimulation with mitogen Con A (10 μg/mL) was included into experiment as a positive control. 24-well tissue culture plates were allowed at 37 °C, 5% CO_2_ incubator for 24 h. Afterwards, the cell suspension was centrifuged at 800× *g* for 10 min at 4 °C and the cell pellet was resuspended in cold PBS pH 7.2 containing 0.5% bovine serum albumin and 2mM EDTA. The IL-10, IL-4, IFN-γ and IL-17 secretion assays were processed according to manufacturer’s recommendation (MACS Cytokine Secretion Assay, Miltenyi Biotec, GmbH, Bergisch Gladbach, Germany). Counterstaining of CD4^+^ T-cells was performed by using rat anti-mouse CD4 FITC conjugate (Antigenix America). 10,000 viable cells were acquired by immunoflowcytometry using a Beckman Coulter FC 500 flow cytometer (Beckman Coulter Inc., Fullerton, CA, USA) equipped with a 488 nm argon laser and a 637 nm HeNe collinear laser, and controlled by CXP software [39]. A lymphocyte gate based on forward scatter vs. side scatter dot plot discrimination and settings was activated prior to further gating to exclude debris. The samples were assayed twice. The values are expressed as percentage of cytokine positive cells among CD4^+^ cells ± SD. Quantitative detection of mouse IL-4, IL-10, IL-17 and IFN-γ cytokines in cell culture media supernatants following PIPOx and Con A specific stimulation of isolated splenocytes (see Section 2.5) was conducted by enzyme-linked immunosorbent assay Mouse Instant ELISA (Thermofisher Scientific, Waltham, MA, USA) according to manufacturer’s recommendations. All samples were measured twice. The data are expressed as average ± SD.

### 2.7. Phagocytosis

Determination of cell phagocytosis, based on the ingestion of FITC-labeled *C. albicans* cells, was assayed under controlled conditions, following incubation with fluorescein isothiocyanate (FITC)-labeled *C. albicans* and RAW 264.7 macrophages for 30 min at 37 °C. Following treatment, the reaction was stopped by ice cooling the samples. The total amount of phagocyting cells, i.e., cells ingested at least one *C. albicans* cell was determined by immunocytometric assay, using a Beckman Coulter FC 500 flow cytometer (Beckman Coulter Inc., Fullerton, CA, USA).

### 2.8. Cell Uptake and Cell Tracking of PIPOx-FITC by Fluorescence Quenching Cytofluorometric Assay

The uptake and intracellular tracking of PIPOx-FITC by RAW 264.7 cells was performed at 37 °C, for time period from 1–24 h, with 0.05, 0.1 and 0.5 mg/mL of PIPOx-FITC. The extracellular fluorescein isothiocyanate fluorescence of PIPOx-FITC has been quenched using 0.4% Trypan blue dye (Sigma-Aldrich, St. Louis, MO, USA). Trypan blue treated RAW 264.7 cells following exposure to PIPOx-FITC were assayed after 30 min incubation in dark by using immunocytometric evaluation. The amounts of adherent extracellular and ingested intracellular PIPOx-FITC were distinguished and determined based on the difference between resulting total number of phagocyting cells and number of phagocyting cells following fluorescence quenching (Trypan blue assay).

### 2.9. Colocalization of PIPOx-FITC in Macrophages

The distribution fluorescently labeled PIPOx-FITC and its colocalization with specific organelles in RAW 264.7 macrophages was assessed by Confocal laser scanning microscope (CLSM) LSM510 META on an Axiovert 200 and 40×/1.2W C-Apochromat objective (Zeiss, Jena, Germany). The optical setup for FITC fluorescence was excitation with 488 nm laser line, and a 500–550 nm long-pass emission filter; for Mito-Tracker-Orange (Zeiss, Jena, Germany) a 543 nm laser line and a 565–615 nm long-pass emission filter, and for LysoTracker-deep red fluorescence. a 633 nm laser line and a 650–710 nm long-pass emission filter. Concentration of PIPOx-FITC for colocalization study was 1 mg/mL in full growth medium. Living cells were treated for 20 h with PIPOx–FITC, then rinsed with PBS and incubated for 10 min with MitoTracker or LysoTracker (both Molecular Probes, Eugene, OR, USA) at concentration of 75 × 10^−9^ mol/dm^3^. Cells were washed with PBS before imaging. A region of interest (ROI) analysis was performed using ZEN software (Zeiss) [40] The results are presented as the average of correlation coefficients from six different cells for each Tracker dye.

### 2.10. Statistical Analysis

The immunobiological results were expressed as mean values ± SD. Data were tested for normality by Shapiro–Wilk test at the 0.05 level of significance. Statistics was performed by one-way ANOVA and post hoc Bonferroni test. Results were considered significant when differences equaled or exceeded the 95% confidence level (*p* < 0.05). Statistics was completed by ORIGIN 2018 software (OriginLab Corporation, Northampton, MA, USA). Pearson’s correlation coefficient has been applied to compare the strength of the relationship between variables.

## 3. Results

### 3.1. Synthesis and Labeling of PIPOx

PIPOx represents a functional polymer with strong potential for different biomedical applications. We showed previously that PIPOx is non-cytotoxic up to a concentration of 10 mg/mL for fibroblasts and macrophages cell lines. For immunomodulation studies, we used splenocytes isolated from Balb/c mice [18]. This paper is a continuation of the work on immunomodulation properties studied on the splenocytes and mouse macrophages [18]. For better comparison, we used for all experiments the same batch of PIPOx as in the previous study. PIPOx was synthesized by the free-radical polymerization using AIBN as an initiator in bulk at 60 °C for 8 h. This type of polymerization provided PIPOx with broader dispersity (Đ = 1.85) and molar mass of 21,000 g/mol. Chemical structure of PIPOx was confirmed by NMR and FTIR (see Figures S1 and S2 in Supplementary Material).

**Figure 1:**
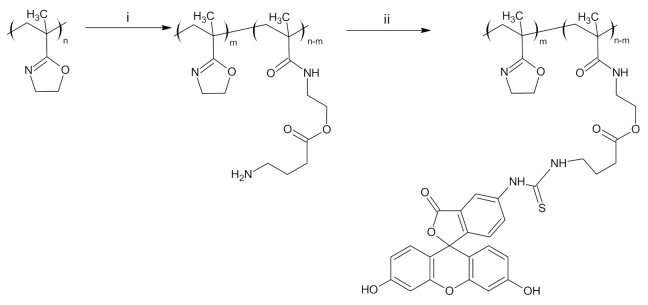
Two-step synthesis of PIPOx labeled with fluorescein (PIPOx-FITC) comprising of the introduction of amino moiety and reaction with fluorescein isothiocyanate (FITC). (i) ABA, DMAc, 120 °C, 6 h, (ii) FITC, 0.1 M bicarbonate buffer, 2–8 °C, 8 h.

**Figure 2:**
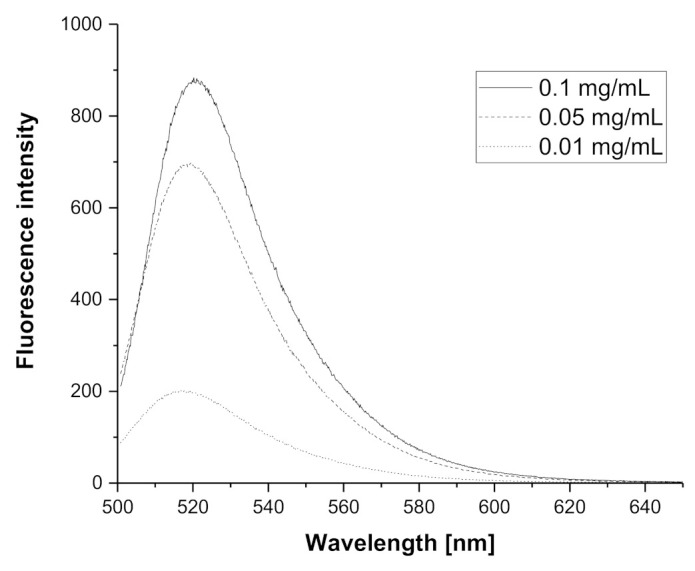
Emission spectra of PIPOx-FITC measured in methanol in three concentrations (0.1 mg/mL, 0.05 mg/mL and 0.01 mg/mL).

Fluorescently labeled PIPOx containing FITC was prepared by two-step modification (Figure 1). The first step included thermally induced reaction of PIPOx with 4-aminobuttyric acid in DMAc at 120 °C for 6 h. The chemical structure was estimated by ^1^H NMR and FTIR (see Figures S3 and S2 in Supplementary Material). Second step comprised addition of FITC on amino groups of PIPOx-ABA according to standard procedure described in Experimental part. Similarly, the real content of fluorescein moieties in PIPOx-FITC was calculated from UV/Vis absorbance and found close to feeding ratio of 1 mol.% (Figure S5 in Supplementary Material). Fluorescence ability of PIPOx-FITC was studied by emission measurements in methanol in different concentrations ranged from 0.01 to 0.1 mg/mL (Figure 2). It can be seen from spectra that emission maximum was for all concentration same and equal to 520 nm. PIPOx-FITC was used for cell internalization and organelles tracking in macrophage cell lines.

### 3.2. Immunomodulation Properties

Immunological behavior of spleen-derived immunocompetent cells and RAW 264.7 cells exposed to PIPOx has been followed up according to inductive release of signature Th1 (IFN-γ), Th2 (IL-4), Th17 (IL-17) and Treg (IL-10) cytokines and effectivity of phagocytic activity.

**Figure 3:**
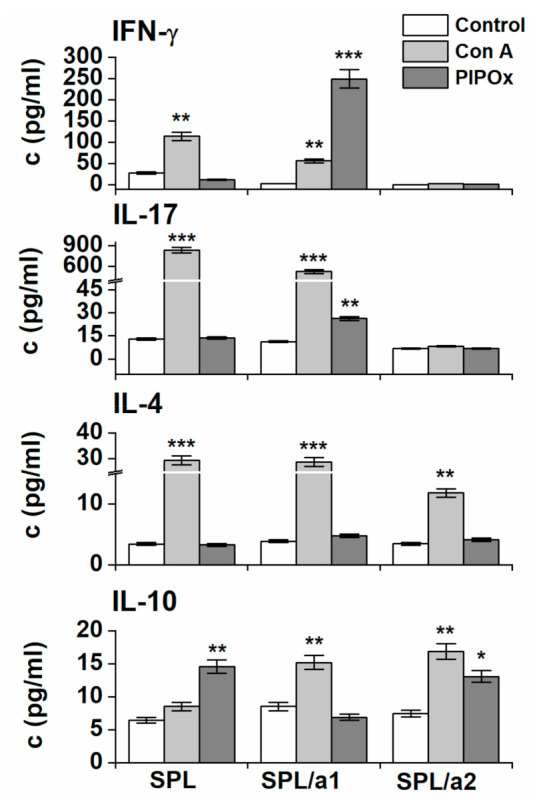
Production of cytokines after in vitro stimulation. Concentrations of IFN-γ, IL-17, IL-4 and IL-10 in media after stimulation of splenocytes and adherent spleen cells after the 1st (SPL/a1) and the 2nd (SPL/a2) adherence period. Response to stimulation with Con A (10 µg/mL) and PIPOx (5 mg/mL). As a negative control, media of unstimulated cells were used. All data are shown as mean ± SD and statistical significance of differences between Con A or PIPOx-stimulated cells and unstimulated cells are expressed: ***—*p* < 0.001, **—0.001 < *p* < 0.01, *—0.01 < *p* < 0.05. Tests were performed in triplicate.

**Figure 4:**
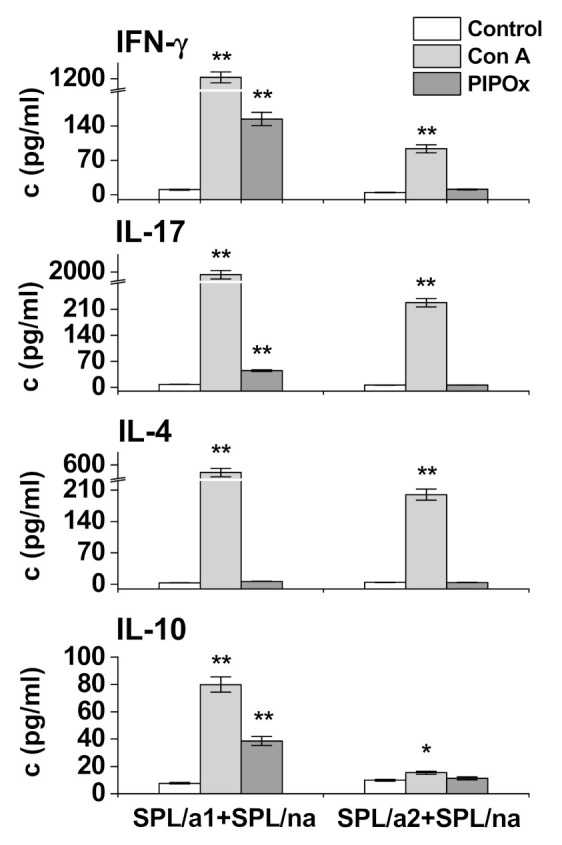
Production of cytokines after in vitro co-stimulation. Concentration of IFN-γ, IL-17, IL-4 and IL-10 in media after co-stimulation of non-adherent spleen cells with stimulated adherent spleen cells obtained after the 1st (SPL/a1) and the 2nd (SPL/a2) adherence period. Response to stimulation with Con A (10 µg/mL) and PIPOx (5 mg/mL). As a negative control, media of unstimulated cells were used. Tests were carried out in triplicate. The experimental data are expressed as geometric means of three replicates ± SD. Levels of significance: ** 0.001 < *p* < 0.01, * 0.01 < *p* < 0.05. Differences were considered significant at 0.01 < *p* < 0.05.

**Figure 5:**
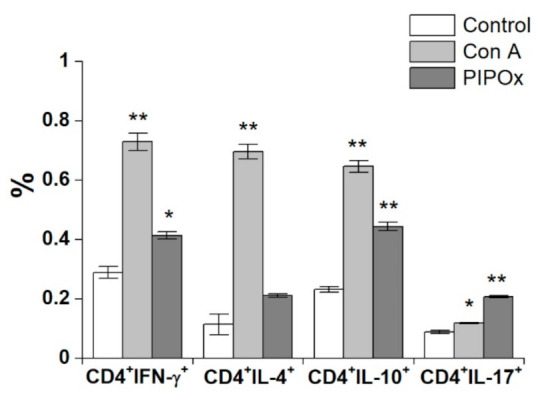
Flow-cytometric analysis of cytokine producing CD4^+^ lymphocytes. Distribution of IFN-γ, IL-4, IL-10 and IL-17 producing CD4^+^ cells within spleen cells stimulated with Con A (10 µg/mL) and PIPOx (5 mg/mL). Tests were done in triplicate. The experimental data are expressed as geometric means of three replicates ± SD. Levels of significance: ** 0.001 < *p* < 0.01, * 0.01 < *p* < 0.05. Differences were considered significant at 0.01 < *p* < 0.05.

The isolated spleen cells were divided into the cell populations using the sequential adherence technique and 4 different groups enriched with distinct types of immunocytes were obtained as described in Table 1. Subsequently, these cell populations were used for stimulation and co-stimulation experiments (Figure 3, Figure 4 and Figure 5).

Cytokine profile analysis of culture media after splenocytes stimulated with PIPOx reveal statistically significant increase of IL-10 production, even higher compared to Con A stimulation. Increase of IFN-γ, IL-17 and IL-4 production was not induced by PIPOx stimulation of complete splenocytes (Figure 3). PIPOx stimulation of adherent spleen cells obtained after the 1st adherence period, enriched in CD11c^+^ and CD14^+^ antigen-presenting cells (APCs), induced significantly enhanced production of IFN-γ (72 times higher compared to unstimulated control and 4.4 times higher than Con A) and increase in production of IL-17 (2.4 times higher compared to unstimulated control but 20 times lower than Con A). The cell-release of IL-4 and IL-10 was not affected. Obtained results indicate direction of the immune response polarization towards Th1/Th17 over Th2 and Treg immune responses (Figure 3). Adherent spleen cells following the 2nd adherence period, more enriched in CD11c^+^ APCs, upon stimulation with PIPOx produced statistically significantly higher amount of IL-10 (1.8 times higher in comparison with unstimulated control although 1.3 times lower than Con A) indicating Treg polarization of immune response (Figure 3).

Adherent spleen cells, enriched in APCs, were pulsed with PIPOx or Con A and co-cultured with non-adherent splenocytes (increased proportion of T-lymphocytes). Culture media after co-cultivation were used for cytokines analysis (Figure 4). PIPOx induced statistically significant increase of IFN-γ production (14.9 times higher than unstimulated APCs, but 8 times lower compared to Con A), IL-17 production (5.7 times higher in comparison with unstimulated APCs although 42.8 times lower compared to Con A), IL-10 production (5.0 times higher than unstimulated APCs and 2.1 times lower compared to Con A) and slight statistically non-significant increase of IL-4 production (1.8 times higher than unstimulated APCs and 79.1 times lower compared to Con A) in cultures containing pulsed adherent spleen cells population following the 1st adherence period (Figure 4). Adherent spleen cells after the 2nd adherence period pulsed with PIPOx did not induced significant increase of cytokines production after co-cultivation with splenic non-adherent cells (Figure 4).

Splenocytes stimulated with PIPOx were analyzed for production of Th1, Th2, Th17 and Treg signature cytokines via flow cytometry (Figure 5). PIPOx exposition induced increase of CD4^+^ producing cells for all monitored cytokines. Results reveal higher percentage of IFN-γ (1.43 times higher than control) and IL-10 (1.92 times higher than control) producing CD4^+^ T-lymphocytes compared to CD4^+^IL-4^+^ (1.85 times higher than control) and CD4^+^IL-17^+^ (2.33 times higher than control) cells (Figure 5). Induction of cytokine producing cells upon stimulation with PIPOx was for IFN-γ, IL-10, and IL-4 lesser than Con A stimulation, except 1.74 times higher proportion of IL-17 producing CD4^+^ cells induced by PIPOx in comparison with Con A stimulation.

PIPOx stimulation of adherent CD11c^+^ and CD14^+^ spleen cells induced significantly enhanced production of IFN-γ and increased production of IL-17 thus indicating polarization of immune response towards Th1 (IFN-γ, *p* < 0.001) and Th17 (IL-17, *p* < 0.01) over Th2(IL-4, ns) and Treg(IL-10, ns) immune responses. Adherent spleen cells more enriched in CD11c^+^ APCs produced significantly higher amount of IL-10 indicating Treg polarization of immune response.

Flow-cytometric analysis of cytokine producing CD4^+^ lymphocytes reveal higher proportion of IFN-γ and IL-10 producing CD4^+^ T-lymphocytes compared to CD4^+^IL-4^+^ and CD4^+^IL-17^+^ cells. Evidently, according to the PIPOx induced production of Th1, Th2, Th17and Treg signature cytokines, pro-inflammatory Th1 (IFN-γ, *p* < 0.001) and Th17 (IL-17, *p* < 0.01) immune responses are more profound in exposed adherent CD11c^+^ and CD14^+^ spleen cells, the adherent spleen cells with higher expression CD11c^+^ exert anti-inflammatory Treg (IL-10, *p* < 0.05) polarized response.

**Figure 6:**
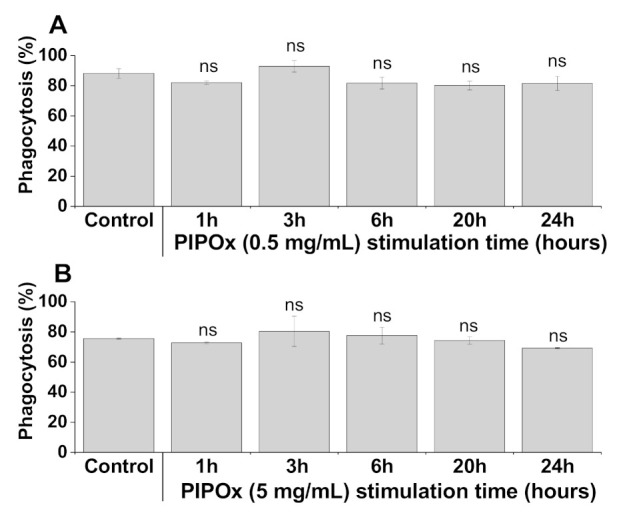
RAW 264.7 macrophage phagocytosis of *C. albicans*–FITC (%) following treatment with PIPOx analyzed by flow cytometry. The phagocytosis of FITC-labeled *C. albicans* cells by RAW 264.7 macrophages was analyzed by pre-treatment of cells for 1, 3, 6, 20, and 24 h with 0.5 mg/mL (**A**), and 5 mg/mL (**B**) of PIPOx and subsequent phagocytosis test analyzed using flow cytometry. Control represents untreated cells (Control). All data are expressed as Mean ± SD. Tests were carried out in triplicate. The one-way ANOVA and post hoc Bonferroni’s tests was used to determine statistical significance of differences between untreated and stimulated cells and is expressed as ns—*p* > 0.05.

The immunocytometric analysis of RAW 264.7 macrophages exposed to different concentration of PIPOx revealed no significant impact on effective phagocytosis of *C. albicans*–FITC complex (Figure 6). Thus, the phagocytic capacity and functional effectivity of RAW 264.7 macrophages remained upon treatment with PIPOx without any significant impacts.

### 3.3. Cell Internalization and Organelles Tracking Study

**Figure 7:**
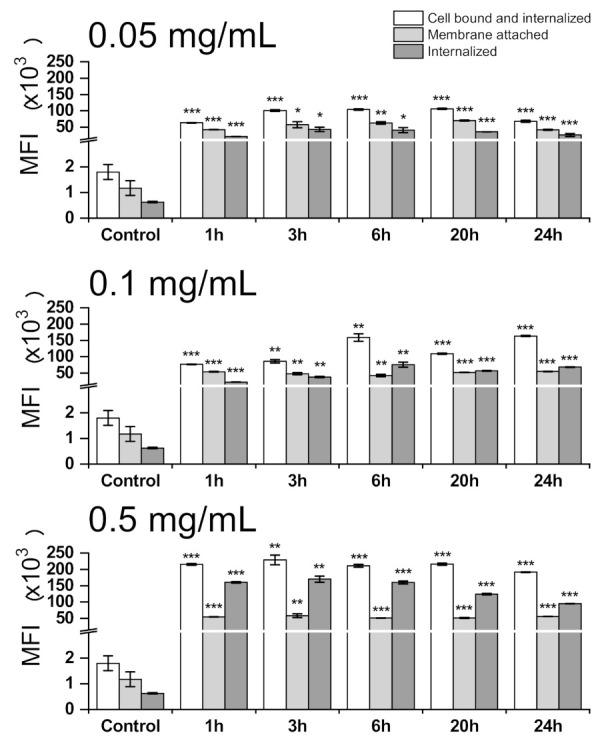
Flow-cytometric analysis of time kinetics and concentration-dependent PIPOx-FITC processing by RAW 264.7 macrophages. The PIPOx-FITC cell bound and internalized by RAW 264.7 macrophages was analyzed by exposition of cells for 1, 3, 6, 20, and 24 h with 0.05 mg/mL, 0.1 mg/mL and 0.5 mg/mL or FITC-labeled PIPOx using flow cytometry. The internalized PIPOx-FITC was analyzed after Trypan blue quenching. The amount of membrane attached PIPOx-FITC is expressed as a difference between the cells with cell bound and internalized PIPOx-FITC and Trypan blue quenched cell population (internalized PIPOx-FITC). Cell bound and internalized, membrane attached and internalized PIPOx-FITC were expressed as mean fluorescence intensities (MFI) of 10,000 analyzed cells. All data are shown as Mean ± SD. Tests were done in triplicate. The statistical significance of differences between control cells and stimulated cells by means of one-way ANOVA and post hoc Bonferroni’s test is presented as: ***—*p* < 0.001, **—0.001 < *p* < 0.01, *—0.01 < *p* < 0.05.

The time and concentration-dependent PIPOx cell-processing (Figure 7) revealed sequential highly concentration-dependent intracellular accumulation of PIPOx. The highest intracellular accumulation of PIPOx for concentration 0.05 mg/mL was observed after 3 h exposition (68.9 times higher compared to the control), for concentration 0.1 mg/mL after 6 h treatment (120.9 times higher compared to the control) and for concentration 0.5 mg/mL after 3 h treatment (271.2 times higher compared to the control). The maximal PIPOx internalization, observed for the highest concentration (0.5 mg/mL), markedly exceeded (3.94-fold) the internalization of the lowest PIPOx concentration (0.05 mg/mL).

**Figure 8:**
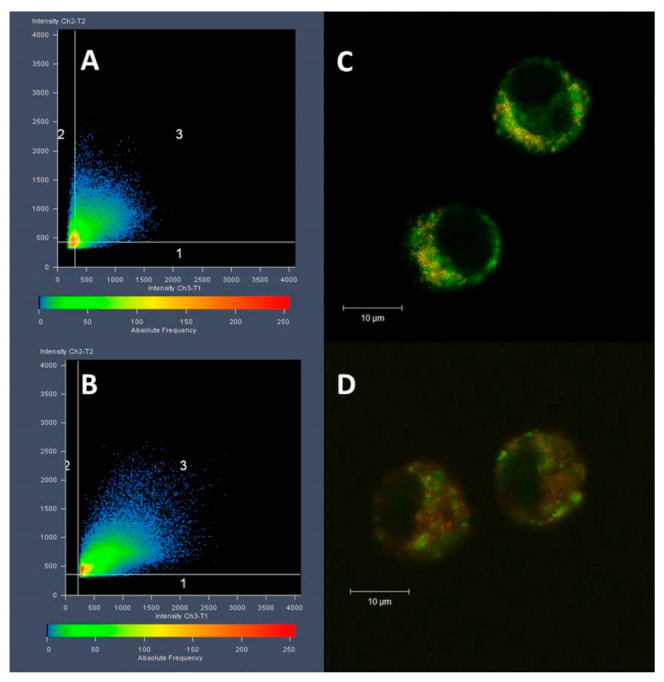
CLSM imaging and ROI analysis of PIPOx-FITC colocalization with organelles. ROI analysis in (**A**) mitochondria, R = 0.3 ± 0.1, and (**B**) lysosomes, R = 0.5 ± 0.16. Merged CLSM images of fluorescently labeled PIPOx-FITC (green) with (**C**) MitoTracker-labeled mitochondria (orange) or (**D**) LysoTracker-labeled lysosomes (red).

Using the flow cytometry, we have clearly demonstrated the uptake of PIPOx into the macrophages. Furthermore, the localization of PIPOx within the macrophage cell was performed using the organelle tracking dyes and fluorescently labeled PIPOx-FITC. The PIPOx-FITC treatment and staining with organelle tracking dyes was followed by ROI analysis according to Pearson and colocalization was expressed by correlation coefficient (R). Colocalization of PIPOx-FITC with organelle is considered when R > 0.5. PIPOx-FITC is localized within the macrophage in vesicular particles as seen on CLSM images (Figure 8). Based on ROI analysis, these vesicles containing PIPOx-FITC do not co-localize with mitochondrial network as R = 0.3 ± 0.1 (Figure 8A,C). Partial colocalization of PIPOx-FITC containing vesicles with lysosomes stained with Lyso Tracker (Figure 8B,D) with R = 0.5 ± 0.16 is observed. There are many vesicles containing PIPOx that do not co-localize with lysosomes.

## 4. Discussion

The assessment of the interaction of the biomaterial structure and modulation of the host immune response represents one of most important strategies for effective in vivo integration of biomaterial. Moreover, immunomodulation strategies based on biomaterials can significantly improve the outcomes of medical implants and tissue engineering therapies. In general, the immunomodulation of immune responses based on the physicochemical modification of a biomaterial is important for effective biomaterial implementation [32]. The current medical research focuses on immunotherapy [32], development of safe vaccines [41] and combinations of therapies [42,43] to achieve the most effective treatment.

Our previous study revealed the immunomodulative properties of PIPOx based on follow-up of the immunocompetent cell proliferation following specific sensitization of splenic cell fractions [18]. The stimulation of splenocytes with PIPOx induced a significant increase of the proliferation rate. Co-cultivation of non-adherent spleen cells with PIPOx-stimulated adherent spleen cells after the first adherence period did not cause an increase in cell proliferation compared with unstimulated control cells, but after coculturing of non-adherent fraction of splenocytes with PIPOx-stimulated adherent spleen cells from a second adherence period, a significant increase of the proliferation rate was observed [18]. These results indicated that PIPOx exerted different immunomodulative effectivity with an emphasis on the phenotype of stimulation-affected immunocompetent cells. Now, regarding these results of previous observations, we perform analysis of the splenic cell-secreted cytokine profile following stimulation, to ascertain character of specific induced immune response and polarization.

In this study, the myeloid phagocytes represented by adherent CD11c^+^ and CD14^+^ spleen cells significantly induced production of IFN-γ and increased production of IL-17 after PIPOx stimulation. Thus, polarization of immune response towards Th1 and Th17 over Th2 and Treg immune responses is indicated. The adherent spleen cells more enriched in CD11c^+^ APCs produced significantly higher amount of IL-10 indicating Treg polarization of immune response. Evidently, in PIPOx-exposed adherent CD11c^+^ and CD14^+^ spleen cells, pro-inflammatory Th1 and Th17 response is more profound, while in the adherent spleen cells with higher expression, CD11c^+^ anti-inflammatory Treg polarized response is applied.

Generally, the Th1 response correlates with protective immunity, contrary to the Th2 responses, whose signature cytokine is IL-4, down-regulating Th1 immunity. Th17 cells were previously believed to be a subset of Th1 cells. They presumably have a regulatory role supporting the Th1-response and down-regulating the Th2-response. Th17 cells producing IL-17 have a crucial role in inflammatory processes. On the contrary, Treg subsets producing signature cytokine IL-10 exerted anti-inflammatory capacity to limit the pro-inflammatory T-cell responses [44].

DCs as APCs respond to molecular patterns by inducing differentiation of naive T-cells into effector T-helper subpopulations and activation of adaptive immunity and initiated both pro-inflammatory and anti-inflammatory immune responses. Moreover, DCs, by taking up, processing, and presenting foreign antigens to Th cells, have a critical role in connecting innate and adaptive immunities. Inflammatory DCs initiate Th17 and Th2 cell responses, while tolerogenic DCs activate Th1 and Tregs. Evidently, the engagement of CD11c^+^ CD14^+^-enriched spleen cells, CD11c^+^-enriched spleen cells, and CD3^+^, CD4^+^, CD8^+^ cells as a result of PIPOx cell-processing, cell presentation, and immune response polarization (Figure 4 and Figure 5) has been observed.

The results revealed that the phagocytic capacity of RAW 264.7 macrophages remained unchanged upon treatment with PIPOx, without down-regulation of cell engulfment and internalization. Previously published immunobiological effectivity of poly(2-ethyl-2-oxazoline) (PETOx) and selected poly [2-(4-aminophenyl)-2-oxazoline-co-2-ethyl-2-oxazoline] (AEOx) copolymer in lymphoid mouse macrophage P388.D1 (Clone 3124) revealed a similar trend of phagocytosis, without any destructive interference with cell viability and phagocyting capability [1].

The uptake of polymers by cells depends on molecular structure, molar mass and the type of cell [45]. High molar mass of polymers can be a limiting factor in cell internalization and therefore some polymer domains that help polymer cell internalization were already identified [46]. In our previous work devoted to cytotoxicity study of PIPOx, we have shown that PIPOx is internalized to fibroblast cells by endocytic pathway [18]. In macrophages, which are professional phagocytes, the phagocytic/endocytic activity is expected. Here, we show that internalization of PIPOx into RAW 264.7 macrophages is concentration-dependent and fast, as after 1h only slow increase of cell-internalized PIPOx is shown during 24 h incubation.

The fate of cell-internalized PIPOx was studied in colocalization studies with organelle markers. As shown by CLSM, PIPOx-FITC is not localized in mitochondria and is localized in vesicular structures that only partially co-localize with lysosomes (Figure 8). This is the difference between PIPOx colocalization in macrophages compared to mouse 3T3 fibroblasts where after 24 h PIPOx was mostly localized in lysosomes [18]. These other vesicular structures in macrophages could resemble other organelles of phagocytic or endocytic pathways, such as phagosomes or endosomes, which supports the hypothesis that PIPOx can be taken up and processed by APCs.

Biomaterials can thus act as synthetic adjuvants or DCs activators. Following internalization of the biomaterial vehicles by DCs, the antigens could be released intracellularly and activate MHC class I and II pathways and induce CD4^+^ and CD8^+^ T-cell immunity [41]. It was shown that polymers can fulfill not only an indirect role as drug carriers [43], agents solubilizing hydrophobic drugs, materials capable of reducing drug cytotoxicity, etc., but they can also function as therapeutic molecules.

It was already mentioned previously that poly(2-oxazoline)s are biocompatible polymers with increasing interest as biomaterials for drug, gene, protein, and radionuclide delivery [2]. For example, novel delivery platforms based on poly(2-oxazolines) with different molar masses have been reported as promising conjugates with interleukins and growth factors [47,48].

They are, however, relatively new in comparison to other classes of water-soluble polymers already established for such use. Besides intensive study of poly(2-oxazoline)s biological properties, only a limited number of them comprises immunomodulatory activity (mainly our previous studies) [1,49,50]. Although PIPOx is formally a member of the poly(2-oxazoline) family, it is a structurally different polymer with free 2-oxazoline rings in the side chain, compared to previously studied polymers of this class.

It was of high importance to subject PIPOx, as a promising biomedical formulation, for evaluation of immunomodulative effectivity in primary immunocytes and its capability to effectively polarize immune responses, thus to reach the best possible strategy to construct new biocompatible delivery systems. Further research in this area is therefore highly appreciated.

## 5. Conclusions

We have shown that PIPOx stimulation of different immunocompetent cells accelerates cell-specific immune responses. PIPOx-sensitized adherent CD11c^+^ and CD14^+^ spleen cells induced statistically significantly enhanced production of IFN-γ and IL-17, indicating polarization of immune cell response towards Th1/Th17 over Th2 and Treg immune responses. Adherent spleen cell-derived CD11c^+^-enriched APCs produced statistically significantly higher secretion of IL-10, the signature cytokine of Treg phenotype. The time and concentration-dependent PIPOx-FITC RAW 264.7 cell-processing revealed sequential intracellular accumulation of PIPOx. The complex phagocytosis of RAW 264.7 macrophages following PIPOx exposure did not exert significant down-regulation of engulfment and internalization throughout effective cell phagocytosis. Using the colocalization of fluorescently labeled PIPOx and organelle tracking dyes, it was shown that PIPOx after internalization to cell occurs in lysosomes and other vesicular structures of endocytic pathway. The results of this study suggest PIPOx as a biocompatible polymer enhancing protective Th1/Th17 immunity over the Treg immune responses.

## Figures and Tables

**Table 1:** Characterization of cell populations enriched by a sequential adherence method. Differentiation markers of cell populations: CD3^+^-T-cells, CD4^+^-helper T-cells (Th), CD8^+^-cytotoxic T-lymphocytes (CTLs), CD11c^+^-DCs, monocytes, granulocytes, CD14^+^-macrophages, monocytes.

Cell Population	CD3^+^ (%)	CD4^+^ (%)	CD8^+^ (%)	CD11c^+^ (%)	CD14^+^ (%)
Splenocytes	30.8 ± 2.2	22.3 ± 1.8	11.8 ± 2.6	8.1 ± 1.7	5.3 ± 1.3
1st adherence period	16.9 ± 3.1	15.7 ± 2.2	7.0 ± 1.3	23.3 ± 2.3	10.0 ± 3.1
2nd adherence period	14.1 ± 2.9	14.7 ± 3.1	6.5 ± 1.2	35.4 ± 3.4	9.1 ± 2.5
non-adherent cells	33.2 ± 2.1	28.3 ± 3.1	12.4 ± 2.3	5.3 ± 1.2	3.3 ± 0.3

## Data Availability

The data presented in this study are available in Supplementary Materials.

## Supplementary Materials

The following are available online at https://www.mdpi.com/1996-1944/14/6/1371/s1, Figure S1: ^1^H NMR spectrum of PIPOx measured in CDCl_3_. Figure S2: ATR-FTIR spectrum of PIPOx, PIPOx-ABA, and PIPOx-FITC. Figure S3: ^1^H NMR spectrum of PIPOx-ABA measured in CDCl_3_. Figure S4: ^1^H NMR spectrum of PIPOx-FITC measured in DMSO-d_6_. Figure S5. UV/Vis measurements of fluorescein isothiocyanate and PIPOx-FITC. (a) UV/Vis spectra of fluorescein isothiocyanate measured in methanol in the concentration range from 5 × 10^−6^ mol dm^−3^ to 10^−4^ mol dm^−3^. (b) Calibration curves of fluorescein isothiocyanate at 278 and 452 nm. (c) UV/Vis spectra of PIPOx-FITC measured in methanol in the concentrations of 0.5 and 1 mg/mL. Concentration of fluorescein unit in PIPOx-FITC calculated from calibration curve was equal to 1 mol.%.
