# Supplementary material for: Cell-Mediated Immunoreactivity of Poly(2-isopropenyl-2-oxazoline) as Promising Formulation for Immunomodulation
Source: Materials (Basel). 2021 Mar 12;14(6):1371. doi: 10.3390/ma14061371 (PMC7999147; doi:10.3390/ma14061371)
Supplement: Supplementary file 1 [file materials-14-01371-s001.pdf]

materials

Supplementary Materials

Cell-Mediated Immunoreactivity of Poly(2-isopropenyl-2-oxazoline) as Promising Formulation for Immunomodulation

Ema Paulovičová [1], Zuzana Kroneková [2], Lucia Paulovičová [1], Monika Majerčíková [2] and Juraj Kronek [2,*]

[1] Immunol & Cell Culture Laboratories, Department Immunochemistry of Glycoconjugates, Center of Glycomics, Institute of Chemistry, Slovak Academy of Sciences, Dúbravská cesta 9, 845 38 Bratislava, Slovakia ema.paulovicova@savba.sk (E.P.); lucia.paulovicova@savba.sk (L.P.)

[2] Department for Biomaterials Research, Polymer Institute, Slovak Academy of Sciences, Dubravska cesta 9, 845 41 Bratislava, Slovakia; zuzana.kronekova@savba.sk (Z.K.); upolmoma@savba.sk (M.M.)

* Correspondence: Juraj.kronek@savba.sk; Tel.: +421-2-3229-4366

Citation: Paulovičová, E.; Kroneková, Z.; Paulovičová, L.; Majerčíková, M.; Kronek, J. Cell-Mediated Immunoreactivity of Poly(2-isopropenyl-2-oxazoline) as Promising Formulation for Immunomodulation. *Materials* **2021**, *14*, 1371. https://doi.org/10.3390/ma14061371

Academic Editor: Montserrat Colilla, Yury Skorik

Received: 20 December 2020

Accepted: 8 March 2021

Published: 12 March 2021

Publisher's Note: MDPI stays neutral with regard to jurisdictional claims in published maps and institutional affiliations.

Copyright: © 2021 by the authors. Licensee MDPI, Basel, Switzerland. This article is an open access article distributed under the terms and conditions of the Creative Commons Attribution (CC BY) license (http://creativecommons.org/licenses/by/4.0/).

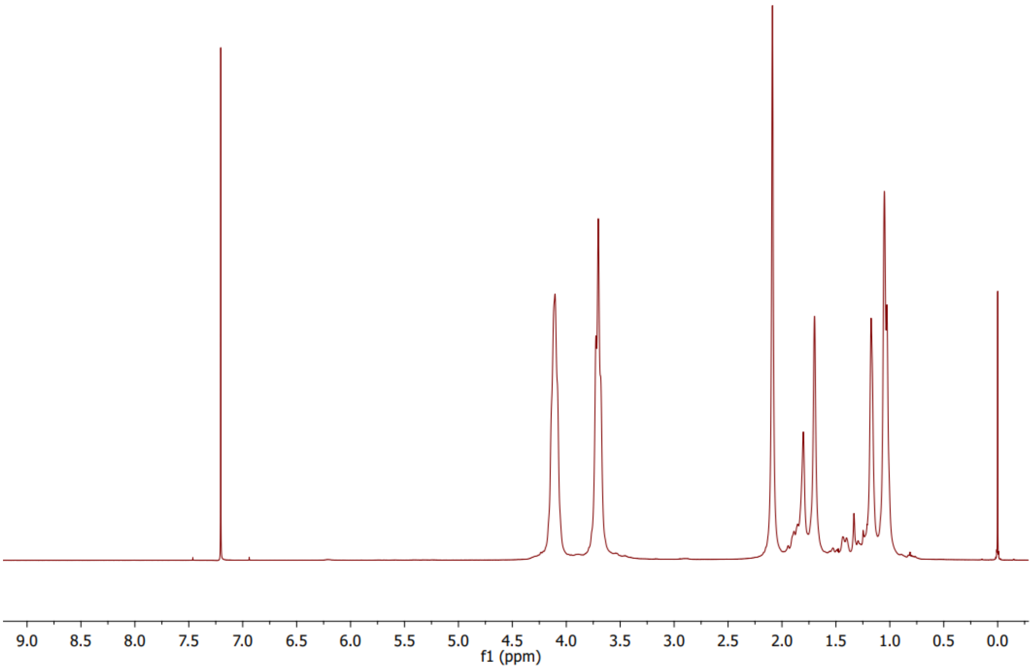

Figure S1. ^{1}H NMR spectrum of PIPOx measured in $CDCl_3$.

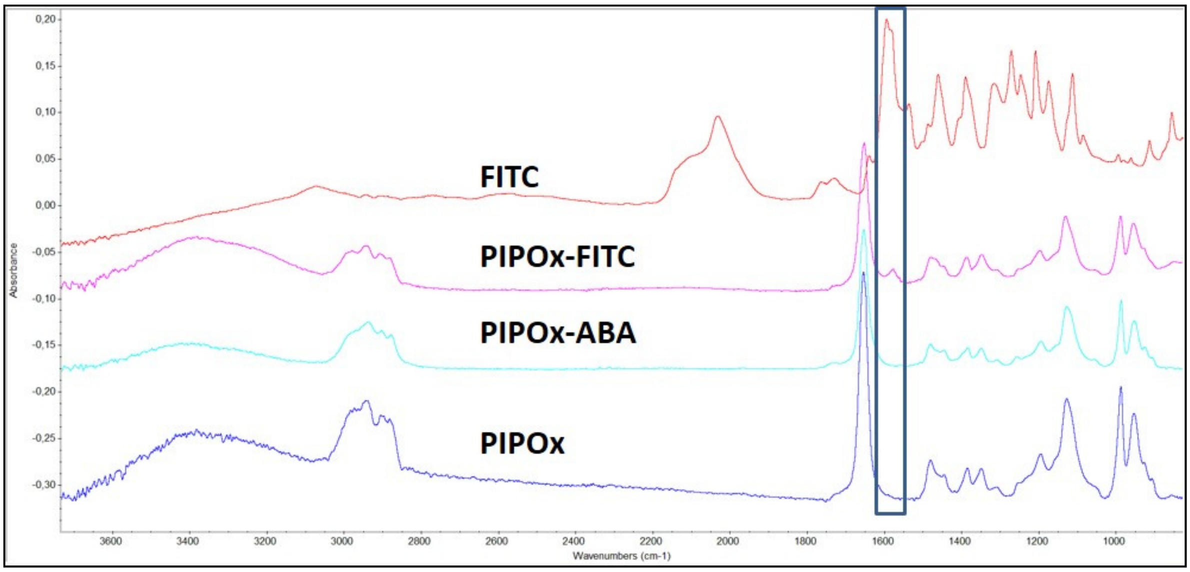

Figure S2. ATR-FTIR spectrum of PIPOx, PIPOx-ABA, and PIPOx-FITC.

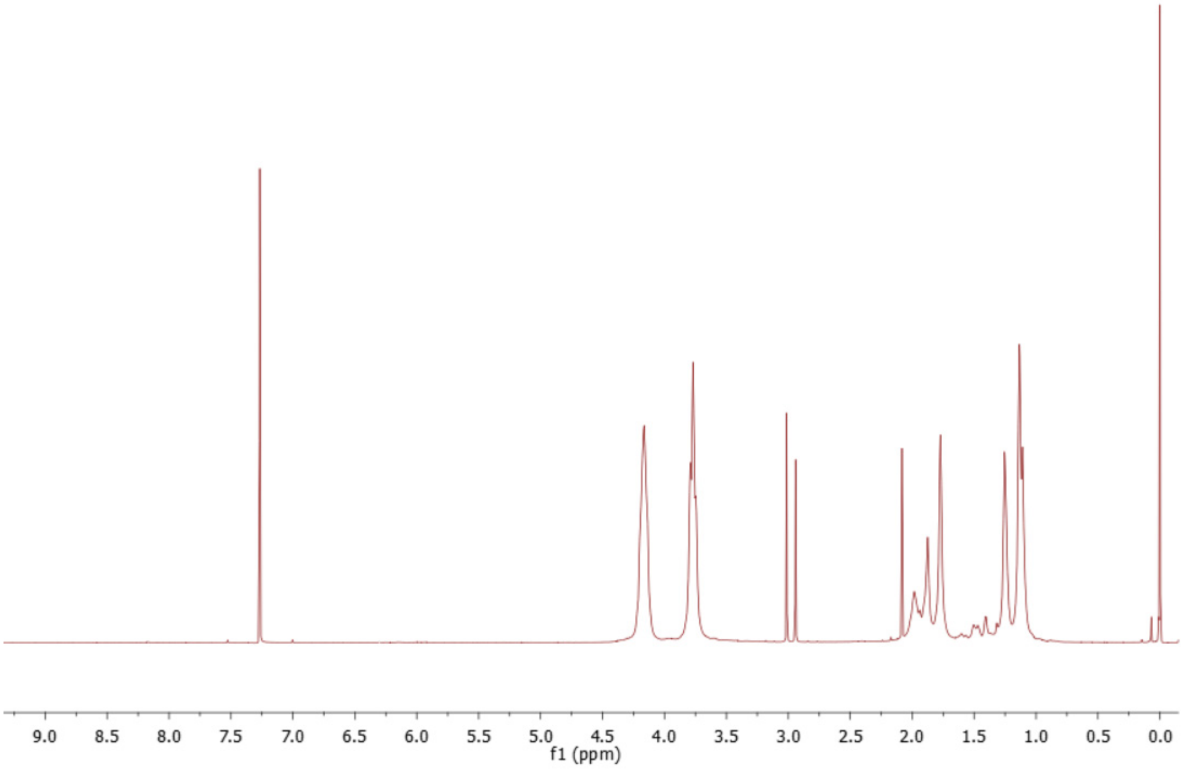

Figure S3. ¹H NMR spectrum of PIPOx-ABA measured in $CDCl_3$.

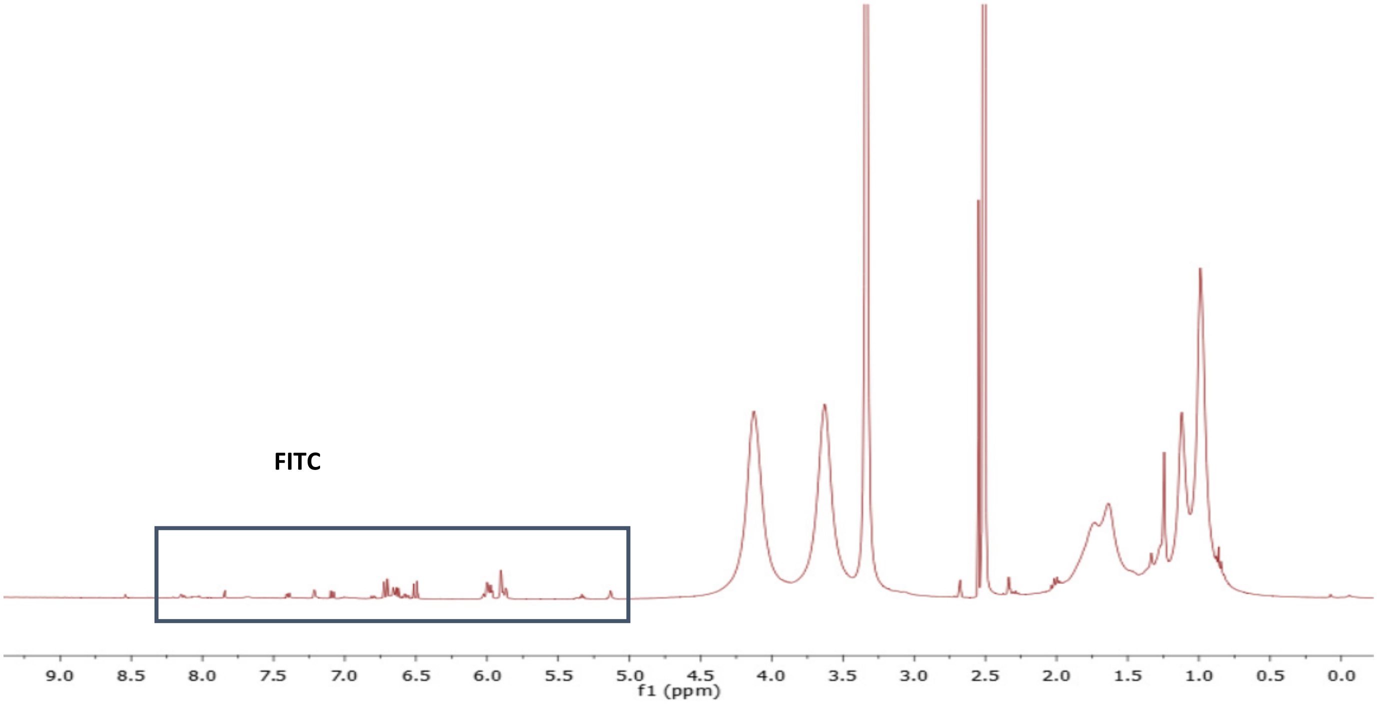

Figure S4. ^1H NMR spectrum of PIPOx-FITC measured in DMSO-d_6.

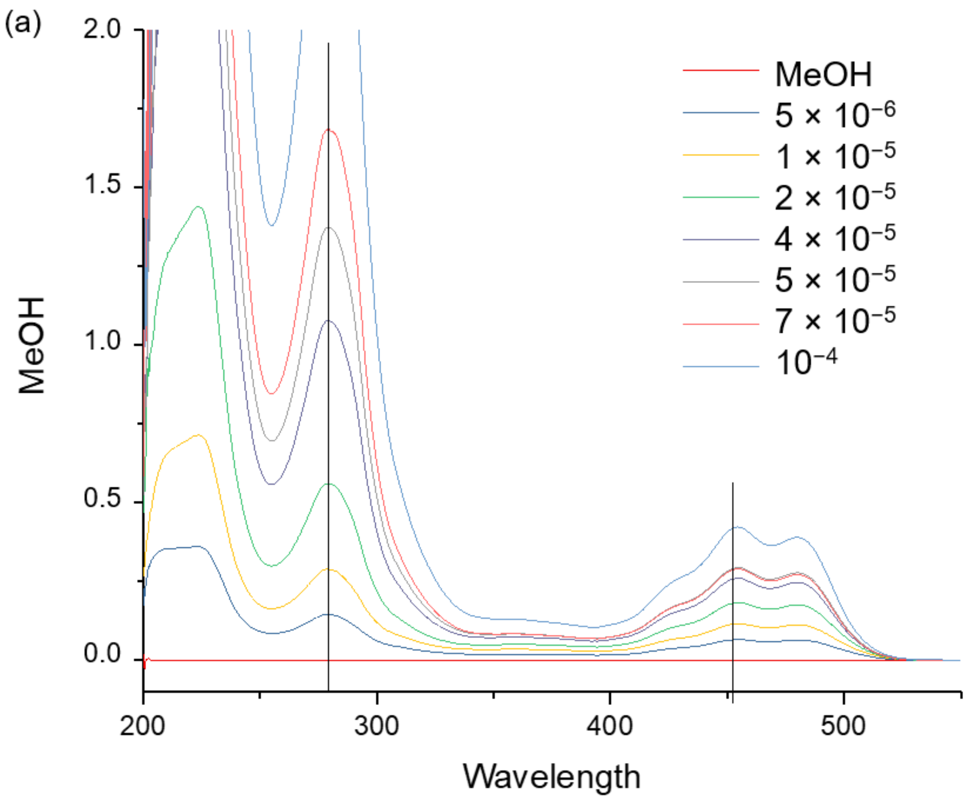

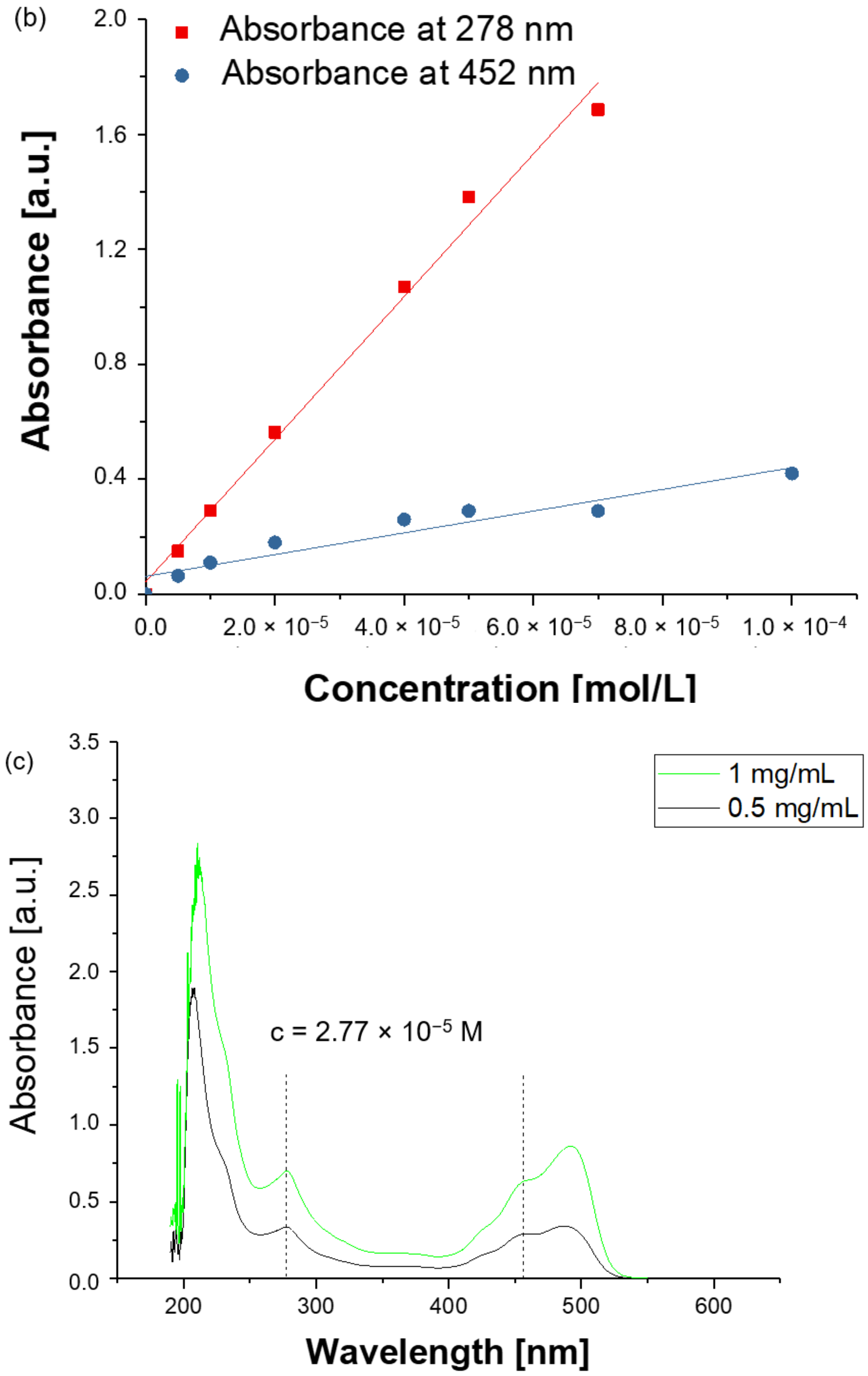

Figure S5. UV/Vis measurements of fluorescein isothiocyanate and PIPOx-FITC. (**a**) UV/Vis spectra of fluorescein isothiocyanate measured in methanol in the concentration range from 5x10^{-6} mol dm^{-3} to 10^{-4} mol dm^{-3}. (**b**) Calibration curves of fluorescein isothiocyanate at 278 and 452 nm. (**c**) UV/Vis spectra of PIPOx-FITC measured in methanol in the concentrations of 0.5 and 1 mg/mL. Concentration of fluorescein unit in PIPOx-FITC calculated from calibration curve was equal to 1 mol %.
